# Effectiveness of orthodontic temporary anchorage devices in canine retraction and anchorage preservation during the two-step technique: a systematic review and meta-analysis

**DOI:** 10.1186/s12903-020-01271-8

**Published:** 2020-10-10

**Authors:** Haonan Tian, Congman Xie, Min Lin, Hongmei Yang, Aishu Ren

**Affiliations:** 1 College of Stomatology, Chongqing Medical University, No.426 Songshibeilu Road, Yubei District, Chongqing, China; 2 Chongqing Key Laboratory of Oral Diseases and Biomedical Sciences, Chongqing, China; 3 Chongqing Municipal Key Laboratory of Oral Biomedical Engineering of Higher Education, Chongqing, China

**Keywords:** Orthodontic implants, Canine retraction, Systematic review, Meta-analysis

## Abstract

**Background:**

Temporary anchorage devices have been used for decades in orthodontic practice for many applications. The aim of this systematic review was to assess the effectiveness of orthodontic temporary anchorage devices in canine retraction during the two-step technique.

**Methods:**

A search was systematically performed for articles published prior to June 30, 2019 in five electronic databases (PubMed, Embase, Cochrane Central Register of Controlled Trials, Web of Science, Scopus). The risk of bias was assessed using the Cochrane risk of bias tool for randomized controlled trials (RCTs) and the risk of bias in nonrandomized studies of interventions (ROBINS-I) tool for controlled clinical trials (CCTs). The Grading of Recommendation, Assessment, Development and Evaluation (GRADE) approach was used for the quality assessment. Data concerning the mean difference in mesial molar movement and extent of canine retraction were extracted for statistical analysis. The mean differences and 95% confidence intervals were analyzed for continuous data. A meta-analysis with a random-effects model for comparable outcomes was carried out.

**Results:**

Three RCTs and five CCTs were finally included. Meta-analysis showed a significant increase not only in anchorage preservation in the implant anchorage group in both the maxilla (1.56 mm, 95% CI: 1.14 to 1.98, *P* < 0.00001) and the mandible (1.62 mm, 95% CI: 1.24 to 2.01, *P* < 0.00001) but also in canine retraction in the implant anchorage group in both the maxilla (0.43 mm, 95% CI: 0.16 to 0.69, *P* = 0.001) and the mandible (0.26 mm, 95% CI: 0.02 to 0.49, *P* = 0.03).

**Conclusions:**

There is very low-quality evidence showing that implant anchorage is more efficient than conventional anchorage during canine retraction. Additional high-quality studies are needed.

## Background

Extracting the premolars and closing the extraction space completely are necessary for orthodontic treatment, especially for patients with bimaxillary protrusion [1, 2]. Additionally, maximum posterior anchorage preservation is crucial for space closure [3–5]. Although transpalatal arches (TPAs) [6], Nance arches [7] and headgear [8] have been widely used for anchorage reinforcement, anchorage loss, mesial inclination of the dental anchorage and molar extrusion, which are undesirable, are still common in orthodontic practice [9–11]. Temporary anchorage devices (TADs) have been used since the last century and have become an alternative reinforcement method to provide anchorage during space closure [12, 13]. Many studies have investigated the efficiency of TADs and have shown that space closure [13–15], tooth intrusion [16] and maxillary expansion [17] can be aided with TADs.

Two-step retraction and en masse retraction are two methods for achieving extraction space closure [18, 19]. Both techniques are efficient for space closure, and there is no significant difference between the two methods in the extent of anterior tooth retraction or molar anchorage loss [5, 18, 20]. However, it takes more time to achieve space closure with two-step retraction than with en masse retraction [5, 21].

A published systematic review [22] compared the difference between en masse and two-step retraction regarding the treatment outcomes and concluded that both methods may lead to similar skeletal improvement, but TADs with en masse retraction can lead to better anchorage control, more anterior retraction and a better facial profile. Many studies [23–25] have shown that en masse retraction with TADs is clinically superior in terms of anchorage preservation, but in orthodontic practice, severe anterior crowding or midline discrepancies may prevent the doctor from performing this procedure [21, 26]. Furthermore, anchorage loss may occur during the initial stage of leveling and aligning [27–29], so early application of TADs may be necessary to retract the canine backward to align the front teeth and attain better anchorage control, especially for cases of severe anterior crowding. Currently, there exists little research on two-step retraction with TADs, and whether it can lead to the same outcome or an even better outcome than en masse retraction with TADs remains inconclusive. Canine retraction is the first step of the two-step retraction method [20], the completion of which is crucial for subsequent incisor retraction.

Skeletal anchorage has two forms, direct skeletal anchorage and indirect skeletal anchorage [30, 31]; a previous study [15] indicated that indirect TADs was not significantly different from conventional anchorage in terms of anchorage preservation during canine retraction, whereas other studies have found a positive effect [14, 32, 33] with direct TADs. Therefore, it is unclear whether the mode of TADs has different effects on the issue. Therefore, this systematic review and meta-analysis aimed to compare the potential of TADs and conventional anchorage in terms of anchorage preservation and canine retraction during the initial canine retraction step of the two-step technique.

## Methods

This systematic review was carried out according to the Preferred Reporting Items for Systematic Reviews and Meta-Analyses (PRISMA) statement [34]. The eligibility criteria were based on PICOS, as follows:

- Study design: Prospective randomized and controlled clinical trial. The exclusion criteria were as follows: review articles, animal studies, case reports, lack of a control group, partial canine retraction and en masse retraction of the anterior teeth, and space closure not performed with sliding mechanics.
- Population: Orthodontic patients requiring extraction of the bilateral first premolars and retraction of the canines during the two-step technique.
- Intervention: Miniscrew implants for anchorage preservation during the first phase of the two-step retraction technique.
- Comparison: Conventional anchorage methods for anchorage preservation during the first phase of the two-step retraction technique.
- Outcomes: The primary outcomes were mesial movement of the first molars (anchorage loss) and the extent of canine retraction in both the maxilla and mandible. The secondary outcomes were tipping of the canines and molars and vertical molar movement. All the outcomes were measured in two cephalometric radiographs; one was taken before canine retraction, and the other one was taken after the completion of canine retraction.

### Protocol registration

The protocol for this systematic review was registered on PROSPERO (CRD42019123343).

### Search strategy and study selection

The following electronic databases were searched for published articles with no language restriction prior to June 30, 2019: PubMed, Embase, Cochrane Central Register of Controlled Trials, Scopus, and Web of Science. The following journals were manually screened: European Journal of Orthodontics, Journal of Orthodontics, American Journal of Orthodontics & Dentofacial Orthopedics, and Angle Orthodontist. Four reviewers (THN, XCM, YHM, LM) independently selected the studies, and disagreements were resolved by consensus. The search strategy is summarized in Additional file 1.

### Data extraction

The following data were extracted: study identification, publication data, sample size, age of patients, types of conventional anchorage, implant diameter, length, and location, extent of horizontal and vertical molar movement, change in molar and canine inclination, extent of canine retraction, and treatment duration. Data extraction was independently conducted by four reviewers (THN, XCM, YHM, LM). Differences were resolved by reviewing the included studies until a consensus was reached. If additional information was needed, the authors contacted an author of the study.

### Risk of bias in individual studies and quality of evidence

Four reviewers (THN, XCM, YHM, LM) independently assessed the quality of the included studies. Cochrane Collaboration’s risk of bias tool [35] for randomized clinical trials (RCTs) was used to assess the quality of the RCTs. The studies were evaluated as having a low, moderate, or high risk of bias. If one of the domains (random sequence generation, allocation concealment, blinding of the participants and personnel, blinding of the outcome assessment, incomplete outcome data, selective reporting, and other bias) was assessed to be at a high risk of bias, the study was given an overall score of a high risk. The risk of bias in nonrandomized studies of interventions (ROBINS-I) [36] tool was used for controlled clinical trials (CCTs). The Grading of Recommendation, Assessment, Development and Evaluation (GRADE) approach was used to evaluate the quality of evidence in four domains: strong, moderate, low, and very low. Any disagreements between the reviewers were resolved by consensus.

### Dealing with zero values

In the event a zero value was presented in the included articles (mean + SD), the SD value was changed to 0.01 mm to enable statistical analysis.

### Data synthesis

Meta-analysis was performed only if the studies reported the same outcome measures. Specifically, a meta-analysis of the mean difference in first molar mesial movement (anchorage loss) and canine retraction was carried out. All clinical studies were statistically evaluated, and significance was established at *P* < 0.05. Heterogeneity was tested using the Q and I^2^ statistics, and a score of greater than 50% indicated extreme heterogeneity. The results of the analyses are shown as forest plots. If significant heterogeneity existed in the study, then subgroup or sensitivity analysis was performed, including analysis of the study design, type of conventional anchorage, and type of implant anchorage. A funnel plot was used to assess publication bias (including more than 10 studies). All statistical analyses were completed with The Cochrane Review Manager (RevMan version 5.1).

## Results

### Study selection

**Fig. 1:**
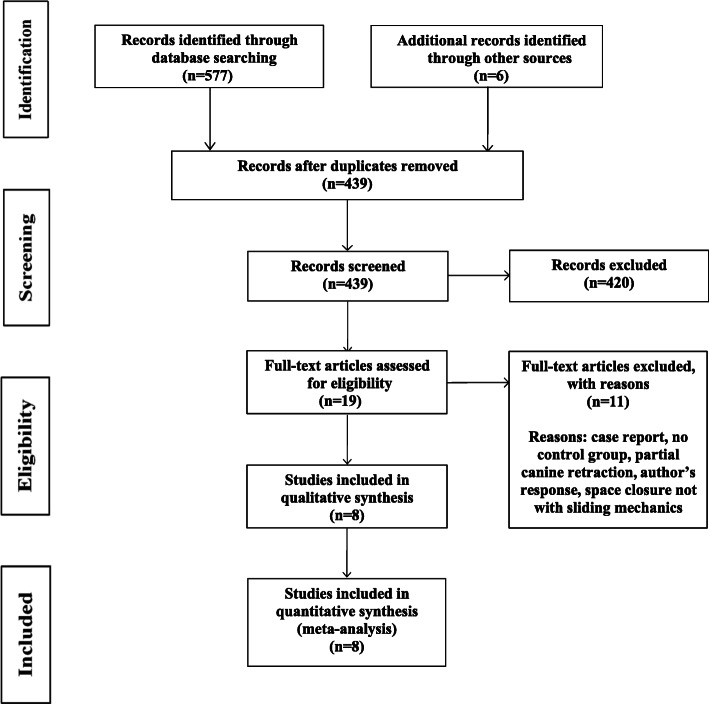
PRISMA flow diagram for the study selection process

The study selection process is illustrated in Fig. 1. A total of 583 articles were identified, and upon review of the titles and abstracts, 420 were excluded, leaving 19 articles. After reading the full texts, 8 studies were included in the present review for qualitative and quantitative synthesis. The 8 remaining studies included three RCTs [14, 32, 33] and five CCTs [15, 37–40]. Information about the excluded records is summarized in Additional file 2.

### Study characteristics

The characteristics of the included studies are presented in Table 1, and the relevant data extracted from the included articles are shown in Table 2. Three RCTs [14, 32, 33] and five CCTs [15, 37–40] were included in the present review. Six studies investigated direct implant anchorage for canine retraction, while two studies used indirect implant anchorage. Four studies [14, 38–40] compared TADs with dental anchorage in a split-mouth study. Two studies [32, 33] compared TPAs with TADs in the maxilla in a parallel study; one [33] also compared lingual bars with TADs in the mandible. The other one [15] compared midpalatal implant-reinforced TPAs with conventional TPAs in the maxilla. One study [37] compared midpalatal implants with dental anchorage in the maxilla and TADs with dental anchorage in the mandible. Three studies [14, 38, 39] inserted implants in both the maxilla and the mandible when the patients’ ANB angle was between 2° and 4° but in only the maxilla when the ANB angle was greater than 5° as a part of camouflage treatment.

**Table 1:** Characteristics of the included studies

Reference	Character of patients	Study type (RCT/CCT)/design	Conventional anchorage	Location of implant	Number of implants	Mode of implant anchorage	Success rate of TAD (%)	Method of measuring tooth movement	Archwire/Force system
Davis et al., 2018 [14]	10(mean age:17.3 years) 6F/4M (10 implant)	RCT/ split-mouth	DA	Placed between the roots of the second premolar and the first molar on the right buccal side (eight patients both in maxillary and in mandible; two patients only in maxillary)	2/1	Direct	100%	cephalometric	19 × 25 SS/ NiTi-closed coil spring
Sharma et al., 2012 [32]	30(mean age:17.4 years) 20F/10M (15 implant;15 non-implant)	RCT/ parallel	TPA	Placed between the roots of maxillary second premolar and first molar on both buccal sides	2	Direct	100%	cephalometric	19 × 25 SS/ Nitinol closed coil springs
Gökçe et al., 2012 [33]	18(mean age:16.7 years) 10F/8M (9 implant;9 non-implant)	RCT/ parallel	TPA (Max) Lb (Man)	Placed between the roots of the first molar and second premolar in all the four quadrants on the buccal sides	4	Direct	100%	cephalometric	NR/ NiTi-closed coil spring
Borsos et al., 2012 [15]	30(mean age:14.22 years) 17F/13M (15 implant;15 non-implant)	CCT/ parallel	TPA	Midpalatal	1	Indirect	100%	cephalometric	16 × 22 SS /NiTi-closed coil spring
Thiruvenkatachari, 2006 [38]	10(mean age:19.6 years) 7F/3M (10 implant)	CCT/ split-mouth	DA	Placed between the roots of the second premolar and the first molar on one buccal side (eight patients both in maxillary and in mandible; two patients only in maxillary)	2/1	Direct	100%	cephalometric	NR/Nickel-titanium closed-coil springs
Hedayati et al., 2007 [37]	19(Implant group:17.4 years DA group:18.2 years) (9 implant,10 non-implant)	CCT/ parallel	DA	Inserted in the midline of the palate approximately parallel to the upper second molars,in maxillary and in the buccal area of the second and third lower molars on two sides in mandible.	3	Indirect (Max) Direct (Man)	81.48%	cephalometric	NR/NiTi pull coil springs
Thiruvenkatachari et al., 2008 [39]	12(mean age:19.7 years) 8F/4M (12 implant)	CCT/ split-mouth	DA	Positioned between the roots of the second premolar and the first molar on one buccal side. (ten patients both in maxillary and in mandible; two patients only in maxillary)	2/1	Direct	100%	cephalometric	16 × 22 SS/ Nickel-titanium closed-coilsprings
Chaudhary et al., 2014 [40]	17 (17 implant)	CCT/ split-mouth	DA	Placed between the roots of the second premolar and the first molar on right buccal side both in maxillary and in mandible.	2	Direct	100%	CBCT generated 2D cephalometric	17 × 25 SS/NiTi closed coil spring

F, Female; M, Male; NR, Not reported; TPA, Transpalatal arch; DA, Dental anchorage; Lb, Lingual bar; Max, Maxillary; Man, Mandible

**Table 2:** Data extracted from the included studies

Reference	Diameter / length (mm) of TISAD^†^	Magnitude of force(g)	Mesial molar movement (mm) ^†^		Tipping of molar(°)		Vertical change of molar (mm) ^†^		Canine retraction (mm) ^†^		Tipping of canine(°)		Treatment time (mon)	
			TISAD (Max/Man)	CA (Max/Man)	TISAD (Max/Man)	CA (Max)/(Man)	TISAD (Max/Man)	CA (Max/Man)	TISAD (Max/Man)	CA (Max/Man)	TISAD (Max)/(Man)	CA (Max) /(Man)	TISAD	CA
RCTS														
Davis et al., 2018 [14]	1.3/8	100	0.1/0.0625	1.3/1.3125	0.30/0.19	2.45/2.69	NR/NR	NR/NR	−4.4/−3.5	−4.2/−3.5	NR/NR	NR/NR	4–7	4–7
Sharma et al., 2012 [32]	1.2/8	150	0/NR	2.48/NR	NR/NR	NR/NR	NR/NR	NR/NR	NR/NR	NR/NR	NR/NR	NR/NR	NR	NR
Gökçe et al., 2012 [33]	1.6/8	100 g	0/0	1.7/1.8	NR/NR	NR/NR	NR/NR	NR/NR	− 4.38/− 4.09	− 3.71/− 3.62	NR/NR	NR/NR	NR	NR
CCTS														
Borsos et al., 2012 [15]	4.1/4	150(cN)	1.57/NR	1.48/NR	NR/NR	NR/NR	NR/NR	NR/NR	NR/NR	NR/NR	NR/NR	NR/NR	9.11 ± 5.70	7.08 ± 4.44
Thiruvenkatachari et al., 2006 [38]	1.3/9	100	0/0	1.6/1.7	NR/NR	NR/NR	NR/NR	NR/NR	NR/NR	NR/NR	NR/NR	NR/NR	4–6	4–6
Hedayati et al., 2007 [37]	2/9(Max) 11(Man)	180	0.58/−0.18	2.5/2.55	NR/NR	NR/NR	−0.330/0	− 0.950/−1.020	NR/NR	NR/NR	NR/NR	NR/NR	5.4 (4–6.5)	5.4 (4–6.5)
Thiruvenkatachari et al., 2008 [39]	1.2/9	100	NR/NR	NR/NR	NR/NR	NR/NR	NR/NR	NR/NR	− 4.2917/− 4.1	−3.7917/− 3.75	NR/NR	NR/NR	4–6	4–6
Chaudhary et al., 2014 [40]	1.2/8	120–150	−0.41/− 0.05	1.31/1.03	NR/NR	NR/NR	NR/NR	NR/NR	−6.75/− 4.83	−6.03/−5.03	−9.51/−7.88	− 6.51/− 4.34	6–8	6–8

NR, Not reported; R, Right; L, Left; Max, Maxillary; Man, Mandible

†For linear measurements, + indicates mesial/occlusal movement and- distal/gingival movement; for angular measurements,+ indicates mesial tipping and- distal tipping

### Risk of bias assessment

**Fig. 2:**
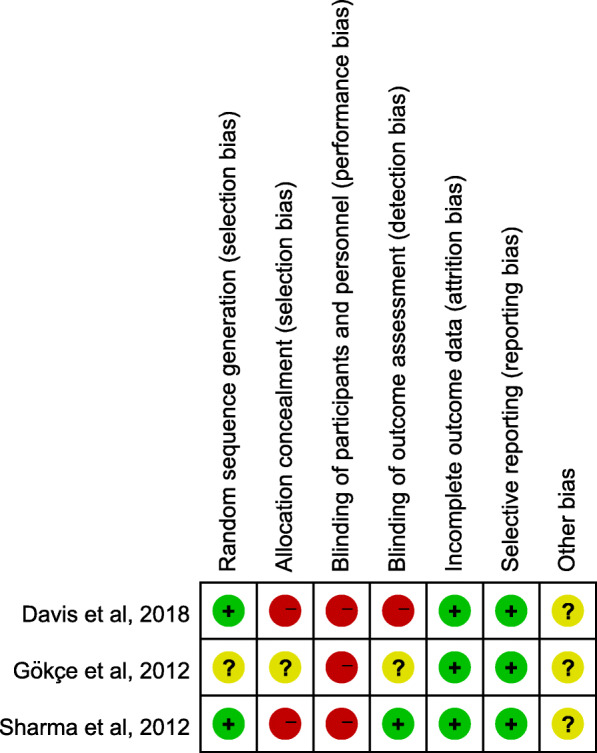
Risk of bias summary for randomized controlled trials

Three RCTs [14, 32, 33] were considered to have a high risk of bias because none of them reported using an appropriate strategy for blinding the participants or personnel. The study by Davis et al., 2018 [14], used a computer-generated program to randomly allocate the sides only, and did not perform allocation concealment. The randomization method used in the study by Sharma et al., 2012 [32], involved random numbers generated by a computer, but the allocation was performed by alternation, which leads to a high risk of bias for allocation concealment. The third study [33] did not report the use of any randomization method, which resulted in an unclear risk of bias. Blinding of the outcome assessment was also difficult in the studies because the TADs could be observed in lateral cephalograms. However, Sharma et al., 2012 [32], removed the miniscrew implants and TPAs before obtaining the cephalometric radiographs that were taken after the completion of canine retraction, resulting in a low risk of bias. Davis et al., 2018 [14], used guide wires to differentiate the right and left sides on the lateral cephalograms, resulting in a high risk of bias. Gökçe et al., 2012 [33], did not report using a process for blinding the assessor, resulting in an unclear risk of bias. The quality assessment results of the RCTs are summarized in Fig. 2.

Five CCTs [15, 37–40] were assessed using the ROBINS-I [36] tool. The study by Hedayati et al., 2007 [37], gave inadequate information regarding the patient inclusion criteria, Thiruvenkatachari et al., 2006 [38], and Thiruvenkatachari et al., 2008 [39], only inserted implants in the maxilla when the ANB angle was greater than 5° as a part of camouflage treatment, which led to a moderate risk of bias in selection. In the measurement of outcomes, Thiruvenkatachari et al., 2006 [38], and Thiruvenkatachari et al., 2008 [39], used wires identifiers, and Chaudhary et al., 2014 [40], used CBCT-generated 2D cephalometric with implants clearly seen on it, which led to a serious risk of bias in the measurement of outcomes. Borsos et al., 2012 [41], used an opaque marker in the approximate position of the implant in both groups, which led to a low risk of bias. Finally, Hedayati et al., 2007 [37], did not present information regarding outcome measurements. Therefore, the overall bias across studies was serious bias in three studies [38–40], moderate bias in one study [37] and low bias in another study [41]. The risk of bias information for the included CCTs is summarized in Table 3.

**Table 3:** Assessment of bias using the Risk of Bias In Non-randomised Studies (ROBINS-I) tool

Authors (years of publication)	Bias due to confounding	Bias in selection of participants into the study	Bias in classification of interventions	Bias due to deviations from intended interventions	Bias due to missing data	Bias in measurement of outcomes	Bias in selection of the reported result	Overall bias
Thiruvenkatachari et al., 2006 [38]	Low	Moderate	Low	Low	Low	Serious	Low	Serious
Hedayati et al., 2007 [37]	Low	Moderate	Low	Low	Low	No information	Low	Moderate
Thiruvenkatachari et al., 2008 [39]	Low	Moderate	Low	Low	Low	Serious	Low	Serious
Borsos et al.,2012 [15]	Low	Low	Low	Low	Low	Low	Low	Low
Chaudhary et al., 2014 [40]	Low	Low	Low	Low	Low	Serious	Low	Serious

### Primary outcome measures

#### Mesial molar movement (anchorage loss)

**Fig. 3:**
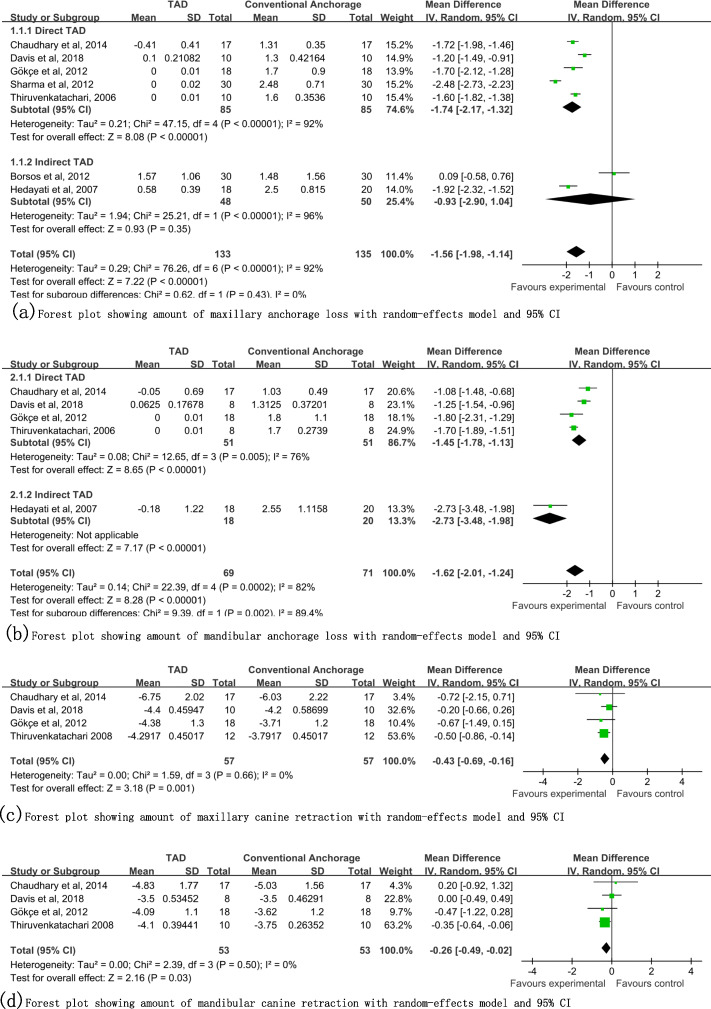
Forest plots of the primary outcomes

Seven studies [14, 15, 32, 33, 37, 38, 40] were qualified for meta-analysis, and the total and subgroup analysis results are given in Fig. 3(a, b). In the maxilla, the results showed a total mean difference of 1.56 mm (95% CI: 1.14 to 1.98), with statistical significance (*P* < 0.00001). Subgroup analysis showed a mean difference of 1.74 mm (95% CI: 1.32 to 2.17, P < 0.00001) in the direct group and a mean difference of 0.93 mm (95% CI: − 1.04 to 2.90, *P* = 0.35) in the indirect group. In the mandible, the results showed a total mean difference of 1.62 mm (95% CI: 1.24 to 2.01), with statistical significance (P < 0.00001). Subgroup analysis showed a mean difference of 1.45 (95% CI: 1.13 to 1.78, P < 0.00001) in the direct group. Only one study [37] included a mandibular indirect group; the results showed a mean difference of 2.73 mm (95% CI: 1.98 to 3.48, P < 0.00001). In both the maxilla and mandible, the direct and indirect groups showed substantial heterogeneity, with I^2^ > 50%.

#### Distal canine movement

Four studies [14, 33, 39, 40] were qualified for meta-analysis of both maxillary and mandibular data, and the results are given in Fig. 3(c, d). In the maxilla, the results showed a total mean difference of 0.43 mm (95% CI: 0.16 to 0.69), with statistical significance (*P* = 0.001); I^2^ = 0. In the mandible, the results showed a total mean difference of 0.26 mm (95% CI: 0.02 to 0.49), with statistical significance (*P* = 0.03); I^2^ = 0.

### Secondary outcome measures

One study [14] included mesial tipping of the maxillary and mandibular molars with direct TADs. The results showed a mean tipping of 0.30° degrees in the TAD group and a mean tipping of 2.45° in the conventional anchorage group in the maxilla (*P* = 0.000); in the mandible, the values were 0.1875° and 2.6875° (P = 0.001), respectively.

One study [37] included vertical displacement of the maxillary and mandibular molars with indirect TADs. The results showed a mean intrusion of 0.33 mm in the study group and 0.95 mm in the control group in the maxilla; in the mandible, the results showed a mean intrusion of 0 mm in the study group and 1.02 mm in the control group. With consideration of the cephalometric error (− 0.55 mm), slight maxillary molar extrusion and mandibular molar intrusion were observed.

One study [40] included distal tipping of the canines in the maxilla and mandible with direct TADs. The results presented tipping of 9.51° in the study group and 6.51° in the control group in the maxilla (*P* = 0.106); in the mandible, the results showed tipping of 7.88° in the study group and 4.34° in the control group (*P* = 0.057).

## Discussion

### Summary of the evidence

Since all meta-analyses included comparable results regardless of the study type (RCT or CCT), the GRADE assessment was processed according to study type for each result. The GRADE recommendations represented very low quality for all of the results. The evidence for all comparisons was determined as being very low quality owing to risk of bias, inconsistency and imprecision. Detailed information is given in Table 4.

**Table 4:** Summary of findings table according to the GRADE approach

Outcomes (study design)	No of Participants (studies)	Quality of evidence (GRADE)	Anticipated absolute effects (95% CI)
Maxillary anchorage loss (randomised trials)	**116** **(3 studies)**	⊕⊝⊝⊝ **VERY LOW**^1^ due to risk of bias, inconsistency, imprecision	**1.8 lower** (2.63 to 0.96 lower)
Mandibular anchorage loss (randomised trials)	**52** **(2 studies)**	⊕⊝⊝⊝ **VERY LOW**^1^ due to risk of bias, inconsistency, imprecision	**1.48 lower** (2.02 to 0.95 lower)
Maxillary anchorage loss (observational studies)	**152** **(4 studies)**	⊕⊝⊝⊝ **VERY LOW**^1^ due to risk of bias, inconsistency, imprecision	**1.39 lower** (1.89 to 0.88 lower)
Mandibular anchorage loss (observational studies)	**88** **(3 studies)**	⊕⊝⊝⊝ **VERY LOW**^1^ due to risk of bias, inconsistency, imprecision	**1.76 lower** (2.41 to 1.1 lower)
Maxillary canine retraction (randomised trials)	**56** **(2 studies)**	⊕⊝⊝⊝ **VERY LOW**^1^ due to risk of bias, imprecision	**0.31 lower** (0.72 lower to 0.09 higher)
Mandibular canine retraction (randomised trials)	**52** **(2 studies)**	⊕⊝⊝⊝ **VERY LOW**^1^ due to risk of bias, inconsistency, imprecision	**0.14 lower** (0.28 lower to 0.57 higher)
Maxillary canine retraction (observational studies)	**58** **(2 studies)**	⊕⊝⊝⊝ **VERY LOW**^1^ due to risk of bias, imprecision	**0.51 lower** (0.86 to 0.16 lower)
Mandibular canine retraction (observational studies)	**54** **(2 studies)**	⊕⊝⊝⊝ **VERY LOW**^1^ due to risk of bias, inconsistency, imprecision	**0.31 lower** (0.6 to 0.03 lower)

All eight studies [14, 15, 32, 33, 37–40] included patients requiring maximum anchorage for space closure. Although there were three articles [14, 38, 39] using different implant strategies, this did not seem to affect the final results, since the mandibular data without implants were not included in the study.

### Summary of the results

Anchorage reinforcement during the space closure stage is a major issue in orthodontic treatment [4]. Conventionally, anchorage is provided by the molar units, a transpalatal arch (TPA), a Nance button or headgear; however, undesirable significant anchorage loss can still occur [42]. TADs are regarded as an ideal alternative method for reinforcing anchorage in en masse retraction, but whether they produce the same effect in canine retraction is inconclusive. The purpose of this systematic review was to evaluate the effectiveness of TADs during canine retraction.

The results of the meta-analysis show that minimizing mesial molar movement is most effectively achieved with TADs. Specifically, overall anchorage preservation of 1.61 mm and 1.62 mm was found in the maxilla and mandible, respectively, which is meaningful compared with that achieved by conventional anchorage methods. However, the results of the subgroup analysis are different. TADs between the second premolar and the first molar on the buccal side cause the molar not to be subjected to any force that will lead to movement. However, indirect TADs in the maxilla had no significant effect on anchorage preservation. The reason may be that the forces in different directions cannot be completely offset, which results in the anchoring teeth being subjected to force, despite indirect reinforcement of the teeth by a steel wire or TPA. The use of indirect implant anchorage is controversial. Ozkan et al. [43] noted that the form of implant anchorage did not affect the results; however, Jang et al. [44] indicated that an indirectly anchored tooth will move mesially with indirect implant anchorage. Additionally, deformation of the TPA [45, 46] brought about by orthodontic force might be another reason for mesial molar movement in the indirect implant group. There is evidence suggesting that some extent of molar retraction may be achieved with TADs, as reported by Chaudhary et al., 2014 [40], and Hedayati et al., 2007 [37]. However, the purpose of these two studies was not to distalize molars, but only to preserve anchorage. The tight fastening between the molar and implant for indirect implants, which are located more distally [37], and friction between the buccal tube and the archwir e[13] for direct implants may be reasons for molar retraction. Therefore, on the basis of very low-quality evidence, direct implant anchorage reinforcement can be considered clinically significant. However, considering the few published articles, the role of indirect implant anchorage with respect to anchorage preservation during canine retraction remains inconclusive.

In our review, two included studies [15, 37] applied palatal implants to reinforce posterior teeth with or without a TPA for indirect implant anchorage in the maxilla. A TPA with a palate implant may cause a marked foreign body sensation and patient discomfort [46]. Substantial clinical heterogeneity existed in the meta-analysis because the included studies varied significantly in the use of osseointegrated and nonosseointegrated implants, study type, bracket slot sizes, archwires and measurement methods.

Canine retraction is the first step of the two-step technique for space closure [20]. Clinically, the extent of canine movement is of great importance for subsequent incisor retraction, especially for patients with dentoalveolar protrusion. Some studies [14, 33, 40] have measured the rate of canine retraction, but considering that different magnitudes of force are used in canine retraction, only the extent of retraction was included in the statistical synthesis. In particular, 0.41 mm and 0.25 mm of canine retraction in the maxilla and mandible, respectively, was consistent with increased anchorage preservation, and increased canine retraction could be achieved with direct implant anchorage. No studies of indirect anchorage were included in this meta-analysis. Therefore, no conclusions regarding indirect implant anchorage can be drawn. Generally, on the basis of very low-quality evidence, direct implant anchorage could facilitate greater canine retraction.

Anchorage loss may also be accompanied by tipping of the molars, but with direct anchorage using TADs, the retraction force acts directly on the canines and away from the molars during retraction. The molars will not be subjected to any force that may lead to excessive molar tipping.

Vertical molar displacement indicates movement of the molar perpendicular to the occlusal plane, usually with intrusion or extrusion. The mesial tipping and extrusion of a molar may lead to an undesirable change in the vertical dimension of the face, which is crucial for the orthodontic treatment of high-angle patients [47]. Tightened ligation between the implant and tooth may exert force to intrude the molar [37]. The implant does have the ability to intrude molars in open-bite cases with elastomeric traction [48]. Implants with a relatively gingival position are more closely or elastically connected, and indirect implants may have the effect of molar intrusion. An implant between the second premolar and the first molar can act as a direct implant, and a ligature wire engaged between the second premolar and the implant can also act as an indirect implant, as reported by Sharma et al. [32].

Bodily canine movement is important for achieving a class I canine relationship, and tipping is undesirable. Herman et al. [3] indicated that canine tipping is related to the method used to ligate the canines to the archwire, with the most bodily canine retraction, perhaps with slight tipping, being achieved using direct implants. However, the bracket slot, archwire size and residual gap may affect canine tipping the most. The better matched the size, the lesser is the tilt [49]. In terms of biomechanics, the occurrence of bodily movement or tipping depends on the relationship between the center of resistance and the direction of the force, i.e., whether the direction of the forces passes through the resistance center. The results show greater canine tipping in the implant group. However, adjustment of the height relationship between the crimpable hook and implant may allow the desired bodily tooth movement to be achieved [50]. Implant anchorage may allow the direction of the force to vary to adjust the extent of tipping during the process.

## Limitations

The number of studies that could be included was relatively small, and the overall quality of the RCTs and CCTs was very low. In particular, there are few studies regarding indirect implant anchorage, which makes drawing a definitive conclusion impossible. The heterogeneity in the meta-analysis of anchorage preservation was relatively high. Due to the small number of articles, publication bias could not be assessed by funnel plot.

## Conclusions

1. During canine retraction, direct TADs can result in better anchorage preservation and canine retraction than conventional anchorage methods.

2. Very low-quality evidence prevents a credible conclusion from being drawn. Further high-quality studies comparing conventional anchorage and TADs during canine retraction are needed.

## Supplementary information

**Additional file 1.** Searching strategy

**Additional file 2.** Articles Excluded After Full-Text Evaluation Based on Eligibility Criteria

## Data Availability

All data generated or analysed during this study are included in this published article and its supplementary information files.

## Abbreviations

RCTs: Randomized Controlled Trials
CCTs: Controlled Clinical Trials
ROBINS-I: Risk of Bias in Nonrandomized Studies - of Interventions
GRADE: Grades of Recommendation, Assessment, Development and Evaluation
TPA: Transpalatal Arch
TADs: Temporary Anchorage Devices
PRISMA: Preferred Reporting Items for Systematic Reviews and Meta-Analyses
CI: Confidence Interval
SD: Standard Deviation
