# Supplementary material for: Effectiveness of orthodontic temporary anchorage devices in canine retraction and anchorage preservation during the two-step technique: a systematic review and meta-analysis
Source: BMC Oral Health. 2020 Oct 10;20:278. doi: 10.1186/s12903-020-01271-8 (PMC7547464; doi:10.1186/s12903-020-01271-8)
Supplement: Supplementary file 1 — Additional file 1. Searching strategy [file 12903_2020_1271_MOESM1_ESM.docx]

| Additional file 1. Searching strategy | | |
| --- | --- | --- |
| Pubmed | ((((((((((((((((headgear) OR lingual bar) OR head brace) OR facebow) OR retraction headgear) OR goshgarian bar) OR nance button) OR transpalatal arch) OR nance arch) OR molar anchorage) OR palatal bar)) OR conventional anchorage)) AND ((((((“Tooth Movement Techniques” [Mesh]) OR “orthodontics”[Mesh]) OR “Orthodontic Anchorage Procedures”[Mesh]) OR “Orthodontic Space Closure”[Mesh])) OR orthodontic)) AND (((((((((((((temporary anchorage device) OR palatal implant) OR screw) OR mini-implant) OR implant) OR micro-implant) OR microimplant) OR miniscrew) OR mini-screw) OR screw implant) OR midpalatal implant) OR miniplate) OR mini-plate)) AND canine | 96 |
| WEB OF SCIENCE | ((((((((((((((((headgear) OR lingual bar) OR head brace) OR facebow) OR retraction headgear) OR goshgarian bar) OR nance button) OR transpalatal arch) OR nance arch) OR molar anchorage) OR palatal bar)) OR conventional anchorage)) AND ((((((Tooth Movement Techniques) OR orthodontics) OR Orthodontic Anchorage Procedures) OR Orthodontic Space Closure)) OR orthodontic)) AND (((((((((((((temporary anchorage device) OR palatal implant) OR screw) OR mini-implant) OR implant) OR micro-implant) OR microimplant) OR miniscrew) OR mini-screw) OR screw implant) OR midpalatal implant) OR miniplate) OR mini-plate)) AND canine | 128 |
| Embase | #1. Canine  #2. headgear OR 'goshgarian bar' OR 'head brace' OR  'retraction headgear' OR facebow OR 'lingual bar'  OR 'nance button' OR 'transpalatal arch' OR  'molar anchorage' OR 'palatal bar' OR 'nance  arch' OR 'conventional anchorage'  #3. 'temporary anchorage device' OR microimplant OR  'palatal implant' OR 'mini implant' OR implant OR  'micro implant' OR miniscrew OR 'mini screw' OR  'screw implant' OR 'midpalatal implant' OR  miniplate OR 'mini plate' OR screw  #4. 'orthodontic anchorage' OR 'orthodontic tooth  movement' OR 'orthodontic space closure' OR  orthodontic OR orthodontics  #5. #1 AND #2 AND #3 AND #4 | 24 |
| Cochrane library | ((((((((((((((((headgear) OR lingual bar) OR head brace) OR facebow) OR retraction headgear) OR goshgarian bar) OR nance button) OR transpalatal arch) OR nance arch) OR molar anchorage) OR palatal bar)) OR conventional anchorage)) AND ((((((Tooth Movement Techniques) OR orthodontics) OR Orthodontic Anchorage Procedures) OR Orthodontic Space Closure)) OR orthodontic)) AND (((((((((((((temporary anchorage device) OR palatal implant) OR screw) OR mini-implant) OR implant) OR micro-implant) OR microimplant) OR miniscrew) OR mini-screw) OR screw implant) OR midpalatal implant) OR miniplate) OR mini-plate)) AND canine | 32 |
| SCOPUS | ( ALL ( headgear  OR  "goshgarian bar"  OR  "head brace"  OR  "retraction headgear"  OR  facebow  OR  "lingual bar"  OR  "nance button"  OR  "transpalatal arch"  OR  "molar anchorage"  OR  "palatal bar"  OR  "nance arch"  OR  "conventional anchorage" ) )  AND  ( ALL ( "orthodontic anchorage procedures"  OR  "tooth movement techniques"  OR  "orthodontic space closure"  OR  orthodontic  OR  orthodontics ) )  AND  ( ALL ( "temporary anchorage device"  OR  microimplant  OR  "palatal implant"  OR  "mini implant"  OR  implant  OR  "micro implant"  OR  miniscrew  OR  "mini screw"  OR  "screw implant"  OR  "midpalatal implant"  OR  miniplate  OR  "mini plate"  OR  screw ) )  AND  ( ALL ( canine ) )  AND  ( LIMIT-TO ( SUBJAREA ,  "DENT" ) ) | 297 |
| Other resources |  | 6 |
