# Supplementary material for: Effectiveness of orthodontic temporary anchorage devices in canine retraction and anchorage preservation during the two-step technique: a systematic review and meta-analysis
Source: BMC Oral Health. 2020 Oct 10;20:278. doi: 10.1186/s12903-020-01271-8 (PMC7547464; doi:10.1186/s12903-020-01271-8)
Supplement: Supplementary file 2 — Additional file 2. Articles Excluded After Full-Text Evaluation Based on Eligibility Criteria [file 12903_2020_1271_MOESM2_ESM.docx]

Additional file 2. Articles Excluded After Full-Text Evaluation Based on Eligibility Criteria

| Reference | Reason for Exclusion |
| --- | --- |
| Ozkan et al, 2016^1^ | Lack of control group |
| Xu et al, 2010^2^ | Lack of control group |
| Martins et al, 2009^3^ | Lack of control group |
| Herman et al, 2006^4^ | Lack of control group |
| Cousley et al, 2015^5^ | Case report |
| Davoody et al, 2012^6^ | Partial canine retraction |
| Wehrbein et al, 1999^7^ | Partial canine retraction |
| Arantes et al, 2012^8^ | Partial canine retraction |
| Upadhyay et al, 2007^9^ | Author’s response |
| Kecik,2012^10^ | Retrospective study |
| Çetinşahin et al, 2010^11^ | Space closure not with sliding mechanics |

References

1. Ozkan S, Bayram M. Comparison of direct and indirect skeletal anchorage systems combined with 2 canine retraction techniques. *American journal of orthodontics and dentofacial orthopedics : official publication of the American Association of Orthodontists, its constituent societies, and the American Board of Orthodontics.* 2016;150(5):763-770.

2. Xu TM, Zhang X, Oh HS, Boyd RL, Korn EL, Baumrind S. Randomized clinical trial comparing control of maxillary anchorage with 2 retraction techniques. *American journal of orthodontics and dentofacial orthopedics : official publication of the American Association of Orthodontists, its constituent societies, and the American Board of Orthodontics.* 2010;138(5):544.e541-549; discussion 544-545.

3. Martins RP, Buschang PH, Gandini LG, Jr., Rossouw PE. Changes over time in canine retraction: an implant study. *American journal of orthodontics and dentofacial orthopedics : official publication of the American Association of Orthodontists, its constituent societies, and the American Board of Orthodontics.* 2009;136(1):87-93.

4. Herman RJ, Currier GF, Miyake A. Mini-implant anchorage for maxillary canine retraction: a pilot study. *American journal of orthodontics and dentofacial orthopedics : official publication of the American Association of Orthodontists, its constituent societies, and the American Board of Orthodontics.* 2006;130(2):228-235.

5. Cousley R. Controlled canine retraction using orthodontic mini-implants coupled with bondable powerarms. *Journal of orthodontics.* 2015;42(4):315-323.

6. Davoody AR, Posada L, Utreja A, et al. A prospective comparative study between differential moments and miniscrews in anchorage control. *European journal of orthodontics.* 2013;35(5):568-576.

7. Wehrbein H, Feifel H, Diedrich P. Palatal implant anchorage reinforcement of posterior teeth: A prospective study. *American Journal of Orthodontics and Dentofacial Orthopedics.* 1999;116(6):678-686.

8. Arantes FM, Kina J, Gonçalves MJB, Gurgel JA, Filho OGS, Santos ECA. Mini-implant and nance button for initial retraction of maxillary canines: A prospective study in cast models. *Dental press journal of orthodontics.* 2012;17(4):134-139.

9. Upadhyay M, Yadav S. Anchorage loss with and without implants during canine retraction. *American journal of orthodontics and dentofacial orthopedics : official publication of the American Association of Orthodontists, its constituent societies, and the American Board of Orthodontics.* 2007;131(1):6; discussion 6.

10. Kecik D. Comparison of temporary anchorage devices and transpalatal arch-mediated anchorage reinforcement during canine retraction. *European journal of dentistry.* 2016;10(4):512-516.

11. Cetinsahin A, Dincer M, Arman-Ozcirpici A, Uckan S. Effects of the zygoma anchorage system on canine retraction. *European journal of orthodontics.* 2010;32(5):505-513.
